# A new approach to precise mapping of local temperature fields in submicrometer aqueous volumes

**DOI:** 10.1038/s41598-021-93374-7

**Published:** 2021-07-09

**Authors:** Alexey M. Romshin, Vadim Zeeb, Artem K. Martyanov, Oleg S. Kudryavtsev, Dmitrii G. Pasternak, Vadim S. Sedov, Victor G. Ralchenko, Andrey G. Sinogeykin, Igor I. Vlasov

**Affiliations:** 1 Prokhorov General Physics Institute of the Russian Academy of Sciences, Vavilov str. 38, Moscow, Russia 119991; 2 Institute of Theoretical and Experimental Biophysics of the Russian Academy of Sciences, Pushchino, Moscow Region, Russia 142292; 3 Wonder Technologies LLC, Skolkovo Innovation Center, Bolshoy blvd. 42, Moscow, Russia

**Keywords:** Biosensors, Biological physics

## Abstract

Nanodiamonds hosting temperature-sensing centers constitute a closed thermodynamic system. Such a system prevents direct contact of the temperature sensors with the environment making it an ideal environmental insensitive nanosized thermometer. A new design of a nanodiamond thermometer, based on a 500-nm luminescent nanodiamond embedded into the inner channel of a glass submicron pipette is reported. All-optical detection of temperature, based on spectral changes of the emission of “silicon-vacancy” centers with temperature, is used. We demonstrate the applicability of the thermometric tool to the study of temperature distribution near a local heater, placed in an aqueous medium. The calculated and experimental values of temperatures are shown to coincide within measurement error at gradients up to 20 °C/μm. Until now, temperature measurements on the submicron scale at such high gradients have not been performed. The new thermometric tool opens up unique opportunities to answer the urgent paradigm-shifting questions of cell physiology thermodynamics.

## Introduction

The invention of the Patch Clamp method^1^ and development of highly sensitive fluorescent Ca^2+^ indicators for imaging of free calcium concentration in the living cell^2^ led to a revolution in our understanding of fundamental mechanisms underlying ionic channels’ functioning. These two outstanding methodological achievements established ultra-local experimental control of the cell membrane potential and intracellular free calcium concentration, both parameters being involved in the equation for electrochemical potential^3,4^. Wide usage of this equation in physiology strongly implies that a distinguishing feature of life manifestation is not only highly organized structural changes in space and time in the living cell, but tuned flows of energy as well. The thermodynamic description of energy flows relies on another parameter of the equation for electrochemical potential, temperature. However, its experimental use as an ultra-local variable for precise temperature control in micro- and nano-volumes in a living cell is still limited by imperfections of current methodological approaches as discussed in Ref. ^5–7^. But not at all by principle theoretical limitations of temperature usage as a macroscopic thermodynamic parameter in nanoscopic watery volumes, where the number of water molecules is still macroscopic. Currently, organic fluorophores^8^, complexes with rare earth metals^9^, biomolecules^10^, thermosensitive polymers^11^, and quantum dots^12^ are used as thermosensors in biological studies. The main problems of such thermosensors are sensitivity to the chemical composition of the local environment, photobleaching, and toxicity.

Here we describe a new practical implementation of ultra-local thermometry based on a specially designed luminescent nanodiamond, which matches well nanoscale thermodynamic requirements in accuracy with no need for intracellular calibration, and allows perfect and easy nanoscale spatial targeting. Only one diamond crystallite is required for use as a thermometer, which practically excludes the toxicity of the thermosensor when applied to living cells. Until now, the diamond thermometry of biological objects was carried out mainly by introducing a large amount of diamond nanoparticles into a cell from a solution^13–16^. The dissimilarity of temperature-sensing characteristics of individual nanoparticles, their random motion and aggregation seriously limit the use of this approach to the precise mapping of local temperature fields in submicrometer volumes. Note that a single diamond crystallite has already been used to measure temperature in a living tissue. However, in those experiments, fairly large diamonds ~ 100 µm, attached to the end of the fiber, were used^17^ and intended to measure some average temperature in the tissue.

It is important to emphasize that the diamond hosting temperature-sensing defects possesses infinite photostability and principled environmental insensitivity, which is not the case with fluorescent molecular thermometers^5–7^.

## Results and discussion

**Figure 1:**
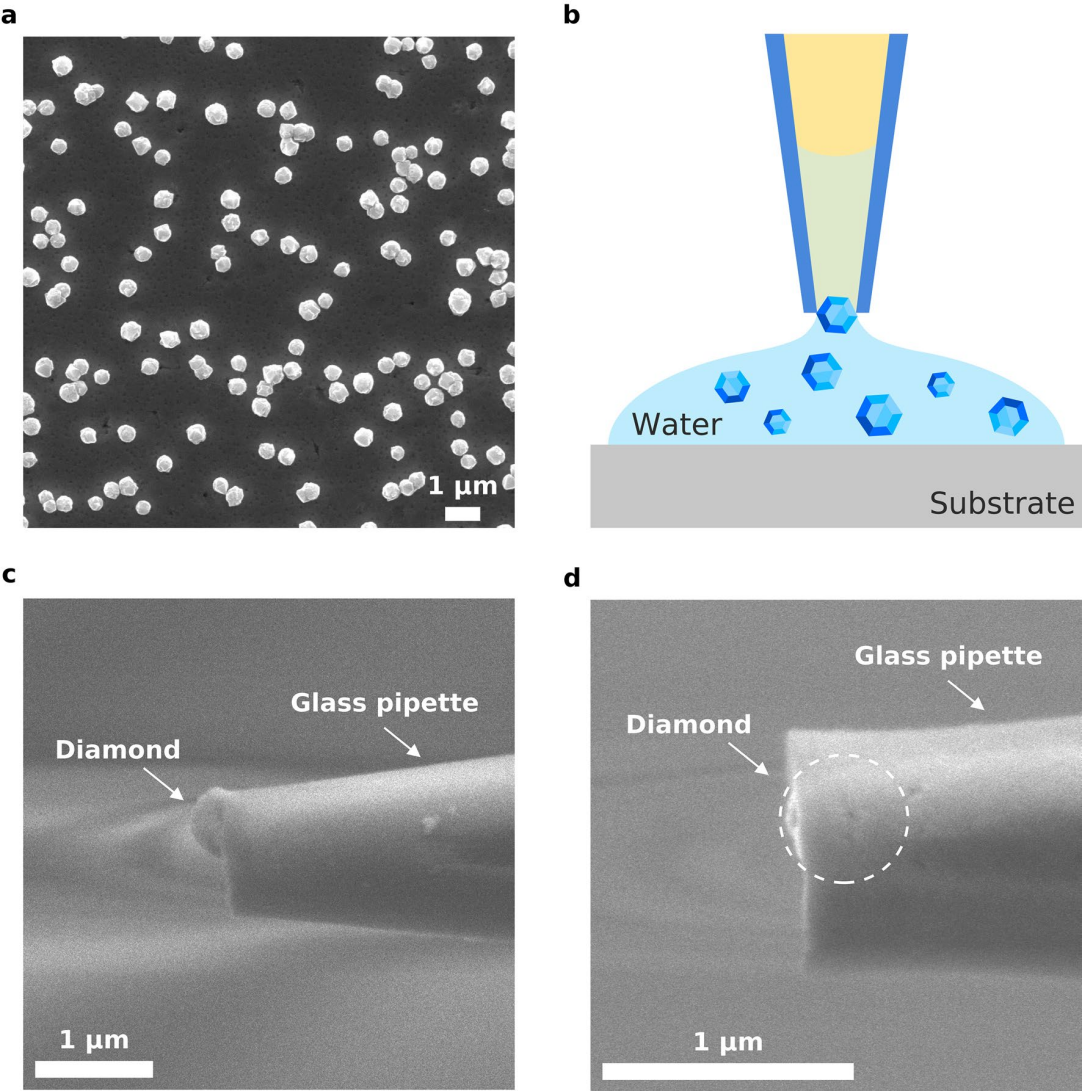
Binding nanodiamond particles to the tip of a glass micropipette: SEM images of a germanium substrate with diamond crystallites synthesized by CVD technique (**a**), schematic representation of diamond drawing into the pipette channel from a water drop (**b**), SEM images of a pipette with a diamond crystallite localized near the entrance (**c**), or at the inlet (**d**) into a capillary.

The main features of the new design of nanodiamond thermometers are as follows. Temperature measurement with submicron spatial resolution is carried out with one luminescent diamond nanocrystal, which is fixed at the entrance of the inner channel of a micro- (nano-) pipette (Fig. 1). To bind the diamond to the tip of the micropipette, a 5-μL drop of distilled water is applied to the substrate with crystallites grown on it (Fig. 1a). At this time, some of the diamond crystallites pass into the aqueous medium. Then, the submicron pipette touches the surface of the drop, and a column of water is drawn into its inner channel under capillary forces (Fig. 1b). When the diamond particle approaches the pipette, due to the good adhesion of the diamond to the glass walls of the pipette, it is likely to adhere either before entering the capillary (Fig. 1c) or at its inlet (Fig. 1d). To securely bind the diamond crystallite to the pipette, the glass tip is slightly melted with an electric heater. The procedure for designing a diamond thermometer is described in more detail in SI. The temperature sensor is precisely positioned in space using a micromanipulator identical to that used in the patch-clamp method. The positioning accuracy is determined by the technical characteristics of the manipulator and in our case is within 50 nm. In this work, we use a 500-nm diamond particle containing luminescent “silicon-vacancy” (SiV) centers. The diamond is produced by a chemical vapor deposition (CVD) technique (see Methods). All-optical detection of temperature is used^18–25^, which is based on the temperature dependence of the spectral position of the maximum of the zero-phonon line (ZPL) of SiV luminescence. The reading and processing of information about the temperature are carried out using a commercial (HORIBA) confocal Raman/luminescence spectrometer and a home-developed software algorithm for determining the temperature by the SiV line position (see Methods).

**Figure 2:**
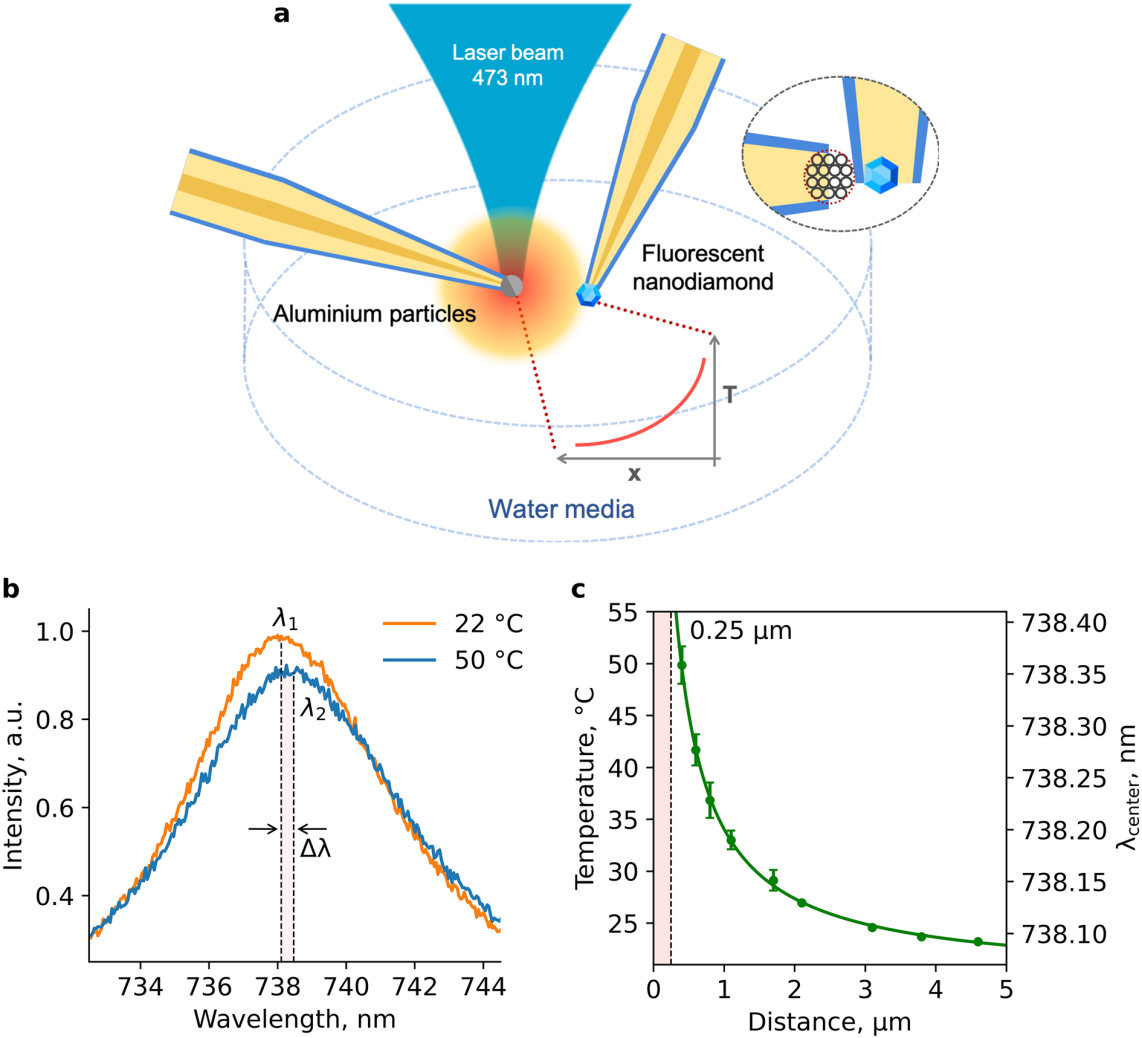
Schematics of temperature distribution evaluation near the local heater: the relative position of the thermometer, heater and laser beam in a cuvette with water (**a**); PL spectra of the diamond thermometer, measured at different distances from the heater, the positions λ_1_ and λ_2_ of SiV ZPL maxima correspond to 22 °C and 50 °C, respectively (**b**); dependence of the SiV ZPL position and the temperature on the distance X between the heater surface and the center of the thermometer, dashed line at X = 0.25 μm corresponds to the distance between the heater surface and the center of the thermometer when they touch each other. The number of measurements in each green point is 7. Corresponding error bars (standard deviations) are minimal (0.3 °C) at distances of 2–5 μm and reach a maximum of 2.5 °C at X = 0.41 μm (**c**).

We demonstrate the submicron resolution of the proposed thermometric instrument to the study of temperature distribution near a local heater, placed in an aqueous medium. The heater is constructed in the same way as the thermometer. For this, a small aggregate of 100-nm aluminum nanoparticles is formed at the entrance of the inner channel of another micropipette, which is precisely positioned in space using a micromanipulator (MM). The use of MM to move the thermometer and heater facilitates the selection of a convenient geometry for the experiment. We excite the SiV luminescence in a diamond crystallite and heat the aluminum particles with one laser beam at a wavelength of 473 nm. Both the thermometer and the heater are positioned at a certain angle to the laser beam and mutually perpendicular to each other in a cuvette with water (Fig. 2a). The continuous wave radiation passes through a water-immersion objective (NA = 0.9) and is focused on the aggregate of aluminum nanoparticles, resulting in its heating. The SiV luminescence is excited at the “tail” of the Gaussian intensity distribution of the laser beam. The position of the heater is fixed and does not change throughout the experiment, ensuring a constant temperature gradient near the heater. The temperature dependence on the distance from the heater is measured by moving the thermometer in steps of 0.2–1 μm along the axis of the pipette with the heater. At each step, a photoluminescence (PL) spectrum is recorded (Fig. 2b), the ZPL is approximated by a Lorentzian-shaped curve using the Levenberg–Marquardt method, and its position is determined (see SI, Section III).

Figure 2c shows the dependence of the SiV ZPL position on the distance X between the heater and the thermometer. Pre-calibration of diamond luminescence against the temperature in a thermostat (see SI, Section IV) allows conversion of the shift in the PL maximum to a local temperature at the particular point. Note the following important features of the experimental data obtained. The 500-nm thermometer:

1. reproducibly “senses” temperature changes of 2.1 °C degrees over 200 nm (near X = 2 µm);
2. is capable of monitoring strong temperature gradients ΔT/ΔX on the submicron scale (the drop ΔT ≈ 15 °C is detected within ΔX ≈ 500 nm near X = 1 µm).

When the two pipettes come into contact, the temperature reaches a maximum value of 50.8 °C. Hereinafter “X” is the distance between the heater surface and the center of the thermometer and at this point ≈ 0.41 μm, which is a sum of the heater pipette wall thickness of ≈ 0.16 μm and a diamond crystal radius of ≈ 0.25 μm, as shown in the inset to Fig. 2a.

The effect of a diamond nanoparticle, which perturbs the temperature field of the heater, on the accuracy of measuring the spatial temperature distribution is analyzed by numerical methods using the COMSOL Multiphysics software package^26^ (comsol.com). The simulated geometry of the "heater-thermometer" system is close to the experimental one. The heater is modeled with an aluminum ball of radius R_1_ = 400 nm. Its size is determined by the inlet diameter of the micropipette inner channel of 800 nm. The thermometer is modeled with a diamond ball of radius R_2_ = 250 nm. Both balls are immersed in a water cube with an edge of 40 μm, the center of which coincides with the center of the heater. The boundaries of this cube are thermally insulated. Note that the glass walls of micropipettes can somewhat affect the temperature field of the heater. However, due to the insignificant difference between the thermal conductivities of glass and water, 1.1 and 0.65 W/m⋅K (ref. ^27,28^), respectively, we neglect this effect and ignore the presence of glass micropipettes in our model (see SI, Section V).

**Figure 3:**
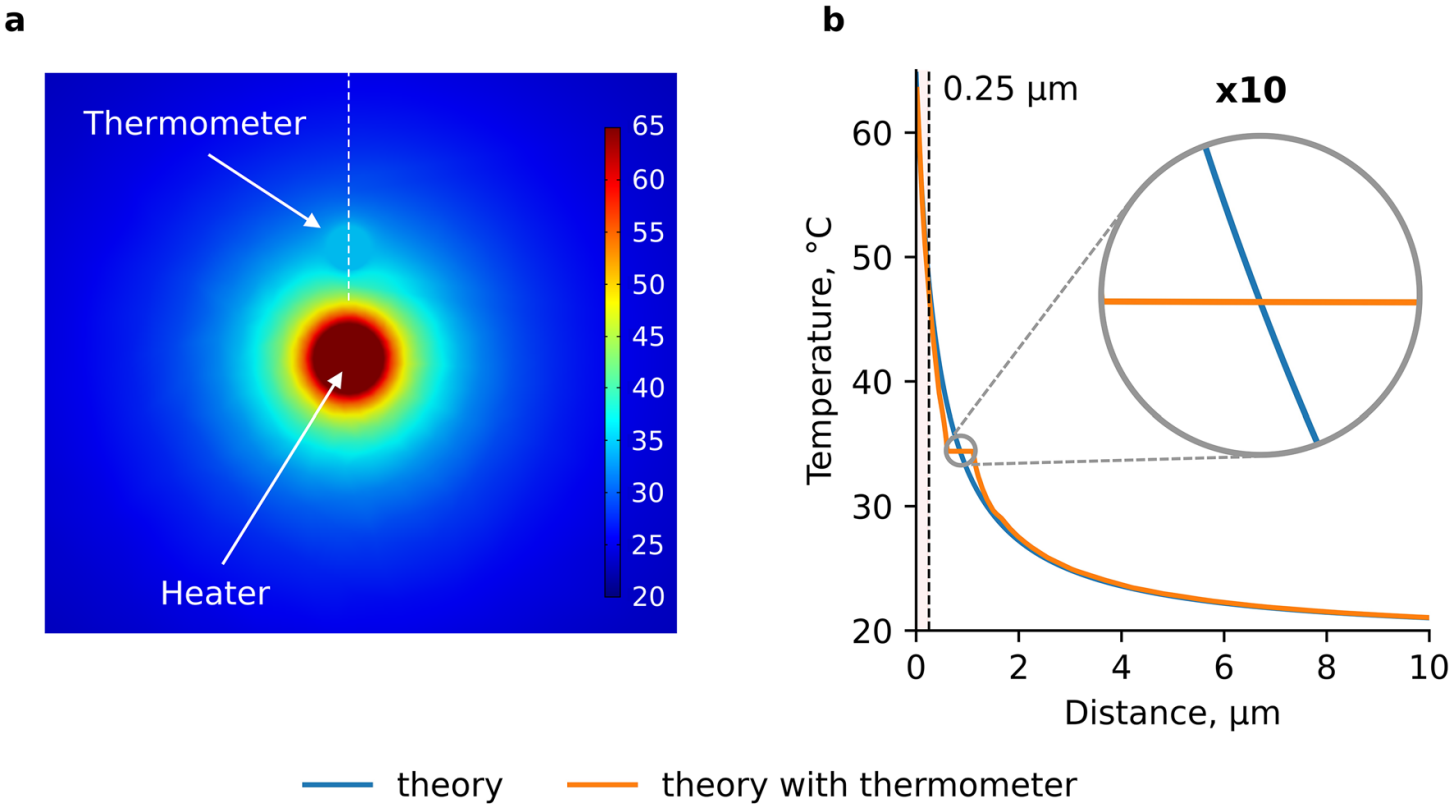
(**a**) A temperature distribution map in the simulated "heater-thermometer" system (COMSOL Multiphysics v. 5.4. www.comsol.com); (**b**) the calculated temperature dependence T (X) without the thermometer (blue curve) and with the thermometer (orange curve) the center of which is located at X = 0.85 μm from the heater surface. The T(X) is calculated along a trajectory, shown by a dashed line in (**a**).

To model the distribution of the temperature field near the heater, it is necessary to know its stationary temperature T_h_ on the heater surface. Its value depends on the laser power, and in our experiments it is T_h_ ≈ 65 °C (see Methods). The spatial distribution of the temperature field of a spherical heat source T (X) is derived from the solution of the stationary heat equation in a spherical coordinate system and is described by the following expression:

1 $${\text{T}} ({\text{X}}) = {\text{T}}_{0} + \left( {{\text{T}}_{{\text{h}}} - {\text{T}}_{0} } \right){\text{R}}_{1} /\left( {{\text{R}}_{1} + {\text{X}}} \right),$$

where T_0_ is the temperature far away from the heater (temperature of the experimental chamber, in our experiment T_0_ is 22 °C), R_1_ is the radius of the heater, X is the distance to the heater surface. The calculated dependence T (X) is shown in Fig. 3b by the blue curve. Placing the diamond thermometer next to the heater introduces some disturbance to the temperature field around the thermometer, as shown in the temperature distribution map (Fig. 3a).

The simulated dependence T (X) in the position of the thermometer at a distance of X = 0.85 μm from the heater is shown in Fig. 3b with an orange curve. The temperature is calculated along a trajectory, shown in Fig. 3a. The T (X) distribution deviates from the blue curve near the thermometer towards the lower T in front of the thermometer and towards the higher T behind the thermometer. Inside the thermometer the temperature drop is only 0.007 °C. The temperature leveling inside the thermometer is explained by the extremely high thermal conductivity of diamond (~ 20 W/cm⋅K, three orders of magnitude higher than the thermal conductivity of water). The temperatures at a distance of 0.85 μm, calculated in the presence of diamond and without it, practically coincide. Thus, the remarkable property of diamond, its high thermal conductivity, makes it possible to almost exclude the effect of its size on the accuracy of temperature measurement even at temperature gradients as high as 20 °C/μm.

**Figure 4:**
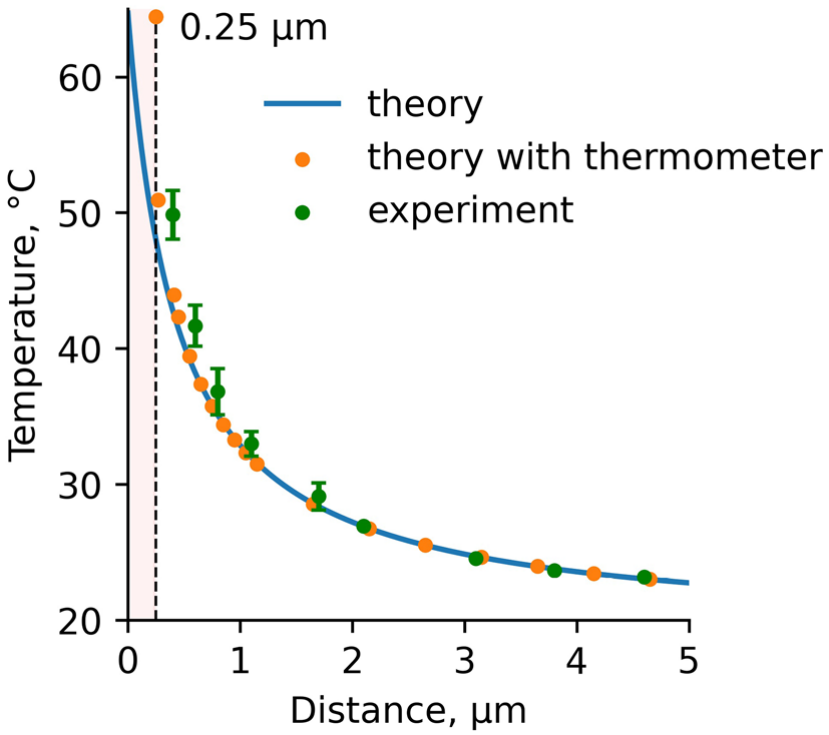
Temperature dependence T (x) without a thermometer (blue curve), calculated thermometer readings (orange dots) and experimental data (green dots). The number of measurements in each green point is 7. Corresponding error bars (standard deviations) are minimal (0.3 °C) at distances of 2–5 μm and reach a maximum of 2.5 °C at X = 0.41 μm.

In Fig. 4 the simulated temperature distribution T(x) in the absence (blue curve) and in the presence (orange dots) of a diamond thermometer, and experimental data, taken from Fig. 2c (green dots), are compared. One can see the appearance of a systematic error in the temperature measurements, as the thermometer approaches the heater, and the temperature gradient grows up. This error is associated with the finite size of the thermometer and a strong temperature gradient (> 20 °C/μm). The systematic error can be significantly reduced by decreasing the size of the thermometer down to 100 nm (see SI, Section VI). The difference between calculated and experimental thermometer readings could be explained by some inaccuracy in the definition of the diamond size and the heater temperature as well as by the difference between the real shape of the heater and the thermometer from the spherical shape.

## Conclusion

We have developed a new design of a nanodiamond thermometer, which allows precise mapping of temperature fields near local heat sources in submicrometer watery volumes. The use of a high-precision micromanipulator eliminates the limitation of the spatial resolution in temperature inherent for optical methods and determined by the diffraction limit of the optical instrument. In our diamond nanothermometer, unlike its predecessors, only one diamond nanocrystal is used, which has the best luminescent properties for predetermined diamond size: the smallest line width and the highest intensity of SiV luminescence. Such a crystal is selected from a multitude of individual diamond nanoparticles synthesized by the CVD method in the mode of spontaneous diamond nucleation on a substrate. A high accuracy of temperature measurement on the submicron scale is demonstrated for a 500-nm diamond thermometer and a 800-nm aluminum heater immersed in water. The calculated and experimental values of temperatures coincide within the measurement error at gradients up to 20 °C/μm. Until now, temperature measurements on the submicron scale at such high temperature gradients have not been performed. Note that 500-nm diamonds will provide good accuracy in determining the temperature as long as a characteristic volume of local temperature changes exceeds the diamond size (in our case, the size of such a volume was about 5 μm). To investigate the smaller volumes, we plan to use smaller diamonds (down to 50 nm).

The new nano-thermometric instrument presented in this work opens up unique opportunities to answer the urgent paradigm-shifting questions of cell physiology thermodynamics, such as:

1. Can open ionic channels be really very hot (tens of degrees °C) in densely packed clusters^29^?
2. Can separate mitochondria be warmer than the surrounding cytoplasm (conundrum of hot mitochondria)^30^?
3. Can the local intracellular temperature be really high (“10^5^ gap issue”)^31,32^?

The presence of a temperature gradient near/inside open ion channels of tens of degrees was theoretically predicted by Chen and coworkers^29^. They argued that the acceleration of ions at the transmembrane electric potential leads to the strong Joule heat release near clusters of ion channels. Our tool makes it possible to check this prediction.

It is important to note that using sharp pipettes (100–200 nm) does not lead to cell damage (violation or washing out of the intracellular environment components) and allows carrying out experiments for tens of minutes^33^.

Further, combining a heater and a thermometer in one unit allows one to implement ultra-local hot spot control inside living cells (call it nanoscopic Temperature Clamp Method), which opens up unprecedented opportunities for micro- and nano-scale thermal initiation of physiological processes in living cells and modulation of their rates in different intracellular compartments^34,35^.

## Methods

### Growing SiV-luminescent nanodiamonds

Nanodiamond containing SiV centers were synthesized on a < 111 > -oriented germanium substrate in a microwave CVD reactor «ARDIS-100» (2.45 GHz). Individual diamond nanoparticles are grown without substrate seeding, using the spontaneous nucleation effect for the initiation of the nanoparticle growth^36^. The choice of substrate material is explained by the weak adhesion between diamond and germanium, which greatly facilitates the transfer of diamond nanoparticles from the substrate surface into an aqueous medium. The synthesis of diamonds is carried out in a hydrogen-methane (96%:4%) gas mixture, with the addition of 0.1% silane (SiH_4_), at a substrate temperature of 700–800 °C, a constant pressure of 75 Torr, microwave power of 4.5 kW, and the deposition time of 30 min. At this concentration of silane, the maximum SiV luminescence intensity is achieved for individual nanodiamonds. Their characteristic size is 0.5 μm. The content of SiV centers is estimated ~ 10^4^ centers per particle.

### Temperature determination

The temperature of water in the vicinity of the local heater is determined from the position of the SiV luminescence line maximum λ_max_. This characteristic of luminescence is sensitive to temperature changes and is proportional to the cube of its value Δλ ~ T^3^ (ref. ^37^). However, for individual diamonds differing in the concentration and spatial distribution of structural defects and SiV centers, the λ_max_(T) may slightly vary from particle to particle. Therefore, for each nanodiamond selected for the thermometer production, a preliminary temperature calibration is carried out using a thermostat in the range of 20–150 °C in 10 °C steps (SI, Fig. 4S). At each temperature value, a ZPL position is determined by the Levenberg-Marquadt approximation method (SI, Fig. 3S).

The heater temperature is determined experimentally from the reference boiling point of water. For this, the power of the input laser radiation P is increased until air bubbles appear near the capillary with a heater immersed in water. It is found that the heater temperature t_boil_ = 100 °C corresponds to P_0_ = 4.3 mW. Then the power was decreased to P_1_ = 2.4 mW. Considering that the room water temperature t_RW_ = 22 °C, the heater temperature is estimated to be 65 °C.

## Supplementary Information

Supplementary Information.
