# Supplementary material for: A new approach to precise mapping of local temperature fields in submicrometer aqueous volumes
Source: Sci Rep. 2021 Jul 9;11:14228. doi: 10.1038/s41598-021-93374-7 (PMC8270900; doi:10.1038/s41598-021-93374-7)
Supplement: Supplementary file 1 — Supplementary Information. [file 41598_2021_93374_MOESM1_ESM.docx]

**A new approach to precise mapping of local temperature fields in submicrometer aqueous volumes**

**Alexey M. Romshin^1^, Vadim Zeeb^2,*^, Artem K. Martyanov^1^, Oleg S. Kudryavtsev^1^, Dmitrii G. Pasternak^1^, Vadim S. Sedov^1^, Victor G. Ralchenko^1^, Andrey G. Sinogeikin^3^, Igor I. Vlasov^1,*^**

**^1^Prokhorov General Physics Institute of the Russian Academy of Sciences, Vavilov str. 38, Moscow 119991, Russia**

**^2^Institute of Theoretical and Experimental Biophysics of the Russian Academy of Sciences, Pushchino, Moscow Region 142292, Russia**

**^3^Wonder Technologies LLC, Skolkovo Innovation Center, Bolshoy blvd.42, Moscow, Russia**

**^*^- corresponding authors**

**Supplementary Information**

**I. Designing a diamond thermometer.**

The design of a diamond thermometer is based on a luminescent nanodiamond built into the inner channel of a glass submicron pipette. The pipette is made of borosilicate glass by pulling and breaking a heated capillary in a vertical micro forge (puller) Narishige: the cylindrical part of the blank is tapering, forming a submicron capillary, which is subsequently used to embed diamond crystallites.


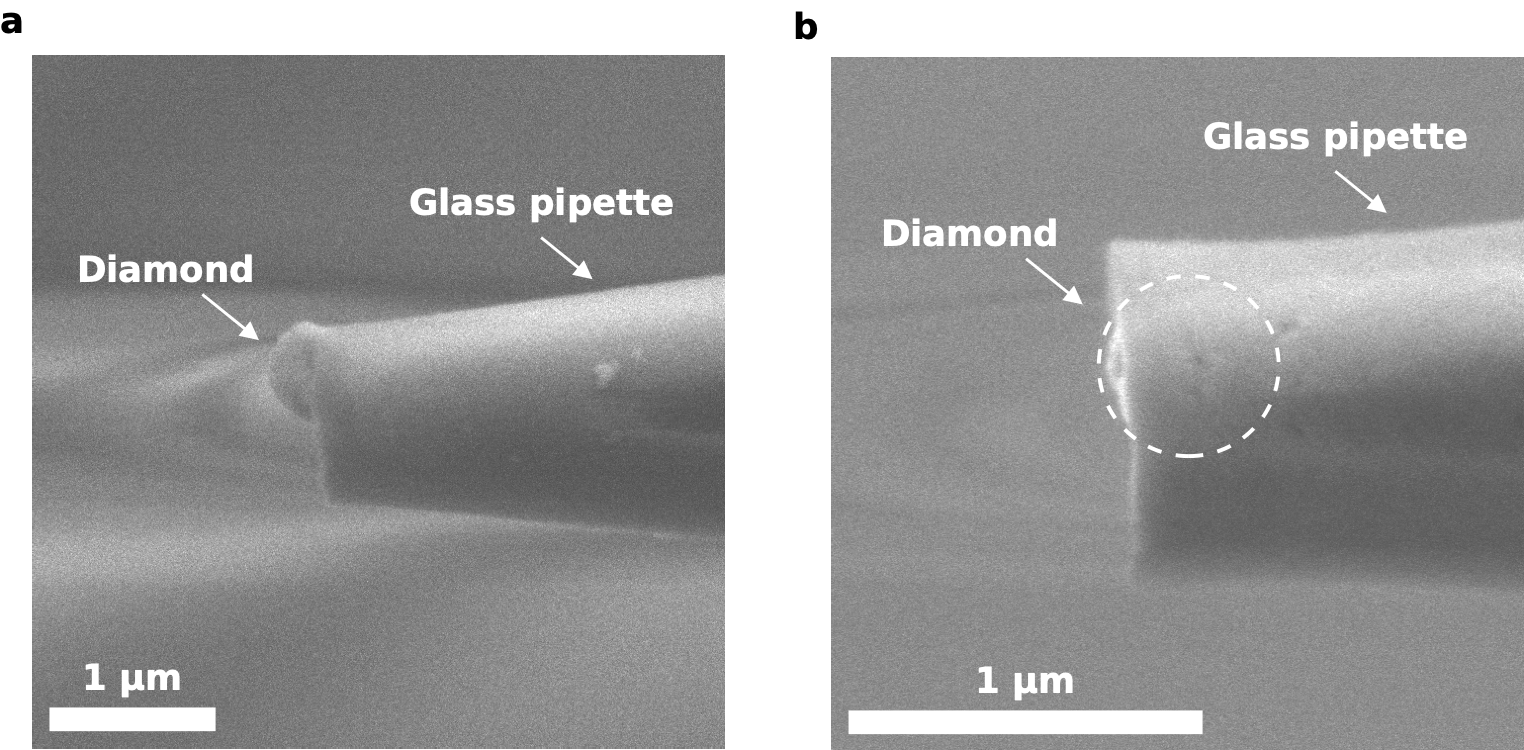


Figure 1S. SEM images of a pipette with diamond crystallite localized near the entrance (a), or at the inlet (b) into a capillary.

A technique for the capture of single diamond nanoparticles by a micropipette capillary from a drop of a nanodiamond aqueous suspension was developed (see Fig. 1). For this, a 5-μL drop of distilled water is applied to the substrate. At this time, some of the diamond crystallites pass into the aqueous medium. At the next stage, a submicron pipette touches the drop surface and a column of water is drawn into its inner channel under the action of capillary forces. The fact that the diamond nanoparticles are captured by the pipette tip is established using optical and scanning electron microscopes (Fig. 1S). The concentration of nanodiamonds in the droplet is insignificant; therefore, under the conditions of our experiment, the probability of several diamond particles entering the capillary is extremely small. Fig. 1S shows two options for successful particle capture with a micropipette. We obtained a series of images in a scanning electron microscope: in four of five pipettes, the diamond was embedded inside the capillary. Thus, the configuration is shown in Fig. 1S (b) represents the most likely configuration for a nanothermometer.

**II. Designing an aluminum heater.**

A powder of aluminum particles with an average size of 50-100 nm and a mass of 2 mg is dispersed in an aqueous medium of 0.5 ml. The formation of a homogeneous aqueous suspension of high-concentration nanoparticles is achieved by cavitation in an ultrasonic bath for one hour. At the next stage, a submicron pipette is brought to the suspension surface and a column of water with aluminum nanoparticles is drawn into the inner channel of the pipette. As a result, an agglomerate of aluminum particles is formed at the entrance to the pipette capillary. SEM image (Fig. 2S) shows that the formed agglomerate is close in shape to a sphere, whose diameter is equal to the capillary size 0.8 µm.


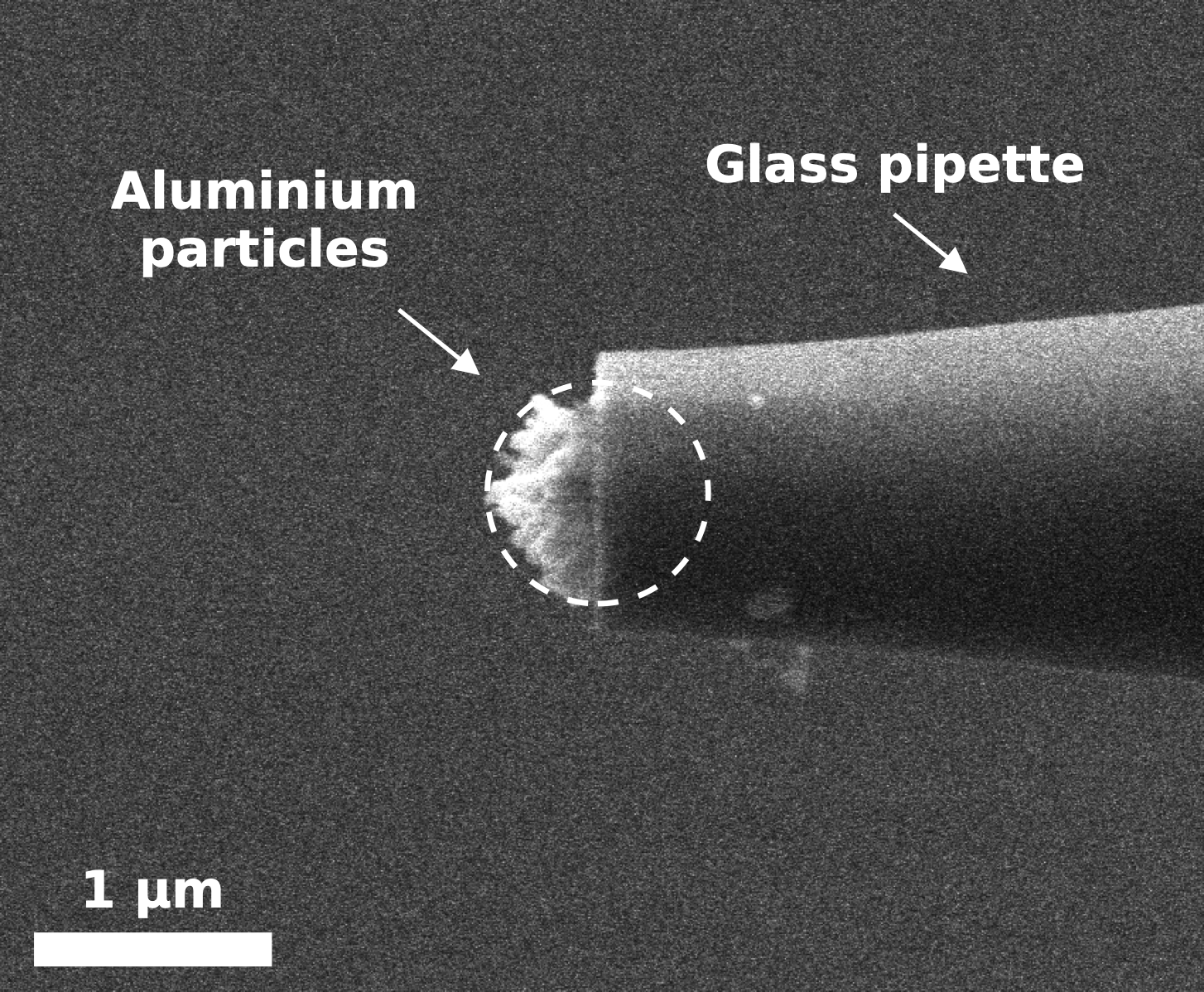


Figure 2S. SEM image of a submicron heater: an agglomerate of aluminum nanoparticles forms at the inlet to the pipette capillary.

**III. Determination of the position of the SiV line.**

A typical luminescence spectrum of SiV-containing nanodiamonds is shown in Fig. 3S. At room temperature, the maximum of the ZPL line is located at a wavelength of ≈738 nm and varies within 0.1 nm from particle to particle. The developed algorithm for determining the line position is based on the approximation of its Lorentzian curve by the Levenberg-Marquadt method. The phonon-related wing, shifted to the long-wavelength region, partially overlaps with the ZPL line, leading to a distortion of its shape. To reduce the effect of such distortion on the approximation precision, we exclude from the approximation the long-wavelength part of the spectrum lying below the level 2/3 of the line intensity maximum I_0_. To reduce the possible influence of additional sources of luminescence emitting at wavelengths <740 nm, for instance, a small broadband luminescence associated with sp^2^ carbon on the surface of CVD diamonds, or narrow-band luminescence at 720 nm often accompanying the SiV luminescence^1^, we exclude from the approximation the short-wavelength part lying below 1/4 I_0_. The approximation of the blue line (Fig. 3S) by the Lorentzian curve allows us to estimate the ZPL maximum position with precision 2∙10^-3^ nm, or 0.15 °C on the temperature scale. This parameter can be significantly improved by increasing the SiV line intensity (for example, using separate lasers to excite the SiV and to heat a local volume), the spectral resolution and sensitivity of the luminescence recording system.


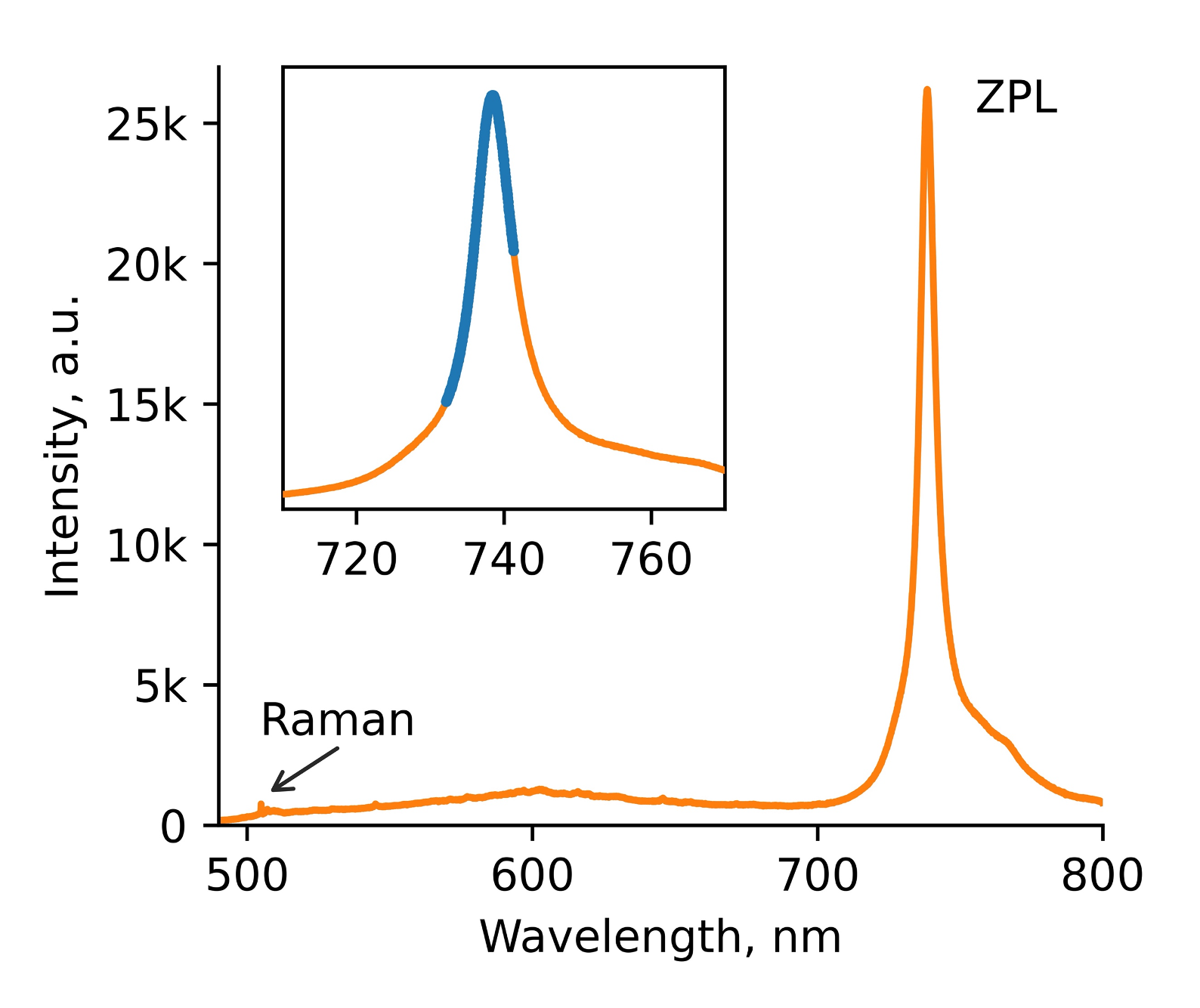


Figure: 3S. Luminescence spectrum of nanodiamonds with SiV centers (orange curve). The part of the spectrum marked in blue in the inset is approximated by the Lorentzian profile.

**IV. Temperature calibration of a SiV-luminescent diamond particle.**

**
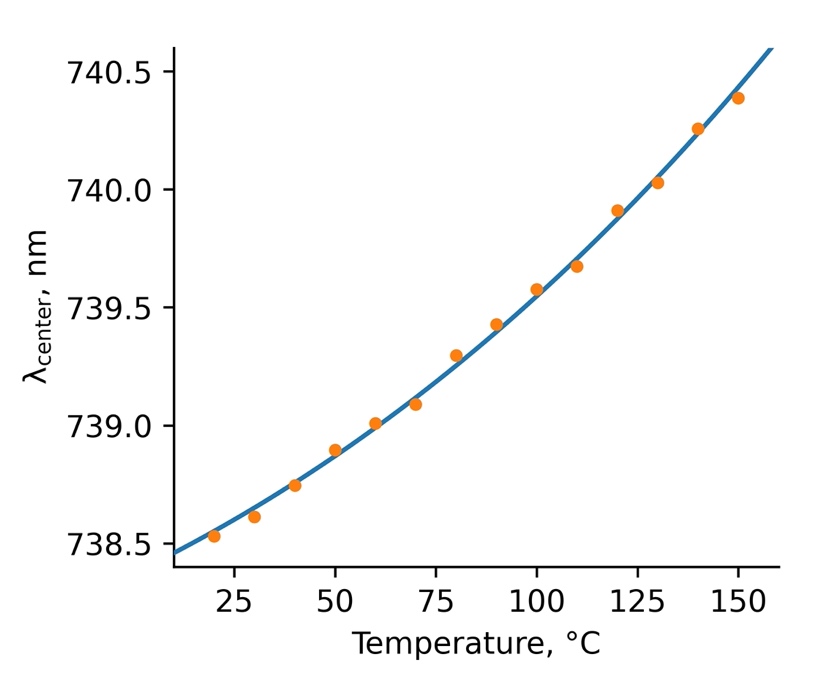
**

Figure: 4S. Calibration curve of SiV luminescence for a diamond particle attached to the micropipette tip (main text, Figure 1d). $\lambda_{max}(T)$ establishes an unambiguous relationship between the SiV line maximum and temperature. Orange dots - experiment, blue curve - approximation. Experimental dependence $\lambda_{max}(T)$ is approximated by the function $a\cdot T^{3}+b$, where $a$ and $b$ are free parameters^37^.

**V. Calculation of T(x) for borosilicate glass capillary.**

Figure 5S. Calculated temperature dependences T (x) without pipette (blue curve) and with 1.1 μm ‎pipette (orange curve) the center of which is located at X = 0.85 μm from the heater surface. The presence of the capillary walls only slightly affects the temperature distribution inside the inner channel of pipette (within 0.2 °C).

**VI. Calculation of T(x) for 100-nm diamond thermometer.**


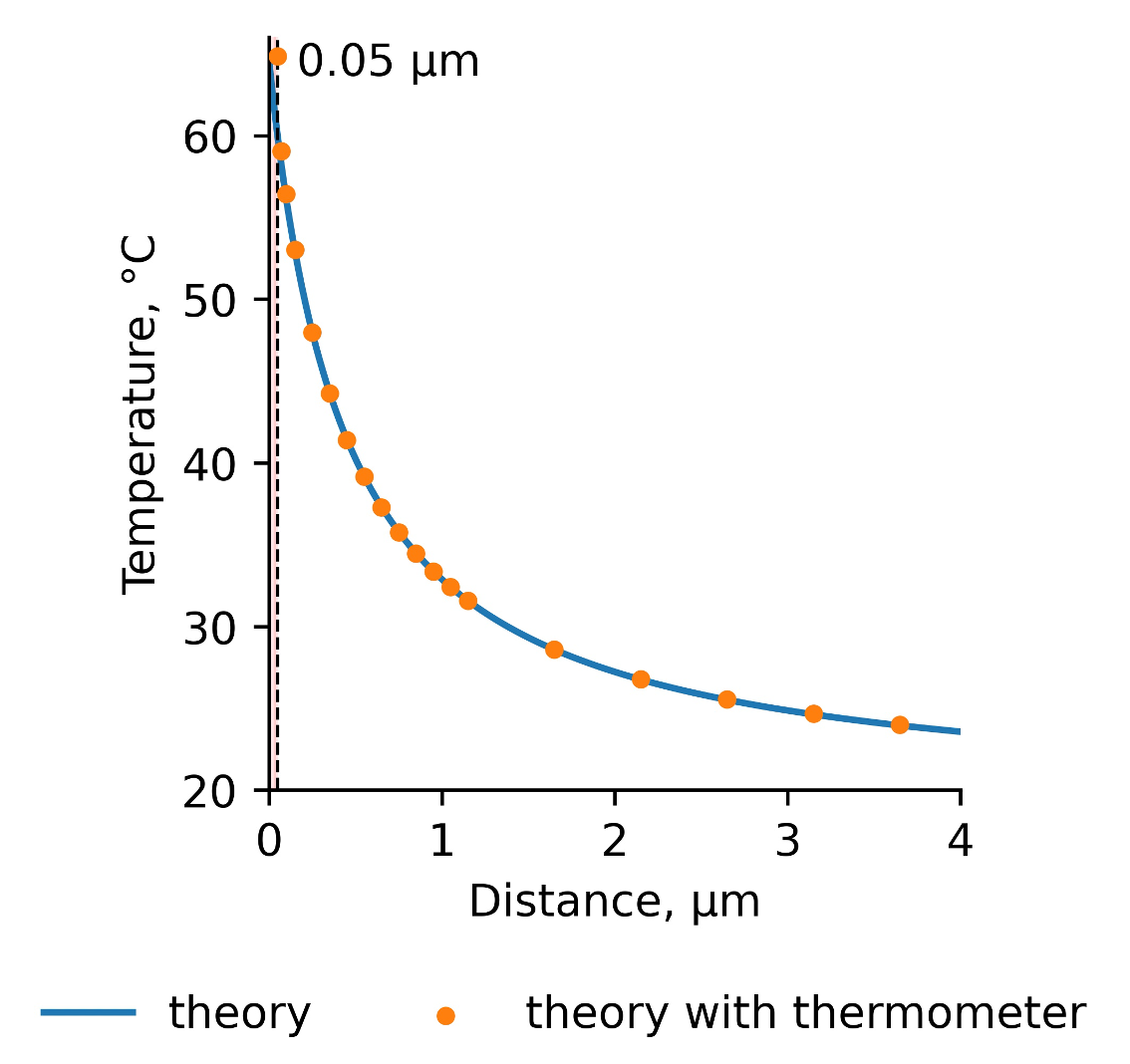


Figure 6S. Calculated temperature dependence T (x) without thermometer (blue curve) and readings of 100-nm diamond thermometer (orange dots).

**References**

1. Sedov, V. S., et al. Color Centers in Silicon-Doped Diamond Films. *Journal of Applied Spectroscopy*, **83(2)**, 229-233 (2016).
